# Microshell Arrays Enhanced Sensitivity in Detection of Specific Antibody for Reduced Graphene Oxide Optical Sensor

**DOI:** 10.3390/s17020221

**Published:** 2017-01-24

**Authors:** Wen-Shuai Jiang, Wei Xin, Shao-Nan Chen, Cun-Bo Li, Xiao-Guang Gao, Lei-Ting Pan, Zhi-Bo Liu, Jian-Guo Tian

**Affiliations:** 1 The Key Laboratory of Weak Light Nonlinear Photonics, Ministry of Education, Teda Applied Physics School and School of Physics, Nankai University, Tianjin 300071, China; jws19860826@126.com (W.-S.J.); xinwei0117@126.com (W.X.); chenshaonan@mail.nankai.edu.cn (S.-N.C.); lcbo710@126.com (C.-B.L.); xiaoguang.gao@foxmail.com (X.-G.G.); plt@nankai.edu.cn (L.-T.P.); jjtian@nankai.edu.cn (J.-G.T.); 2 The 2011 Project Collaborative Innovation Center for Biological Therapy, Nankai University, Tianjin 300071, China

**Keywords:** reduced graphene oxide, microshells, optical sensor, goat anti-rabbit IgG, real-time, label-free

## Abstract

Protein-protein interactions play an important role in the investigation of biomolecules. In this paper, we reported on the use of a reduced graphene oxide microshell (RGOM)-based optical biosensor for the determination of goat anti-rabbit IgG. The biosensor was prepared through a self-assembly of monolayers of monodisperse polystyrene microspheres, combined with a high-temperature reduction, in order to decorate the RGOM with rabbit IgG. The periodic microshells allowed a simpler functionalization and modification of RGOM with bioreceptor units, than reduced graphene oxide (RGO). With additional antibody-antigen binding, the RGOM-based biosensor achieved better real-time and label-free detection. The RGOM-based biosensor presented a more satisfactory response to goat anti-rabbit IgG than the RGO-based biosensor. This method is promising for immobilizing biomolecules on graphene surfaces and for the fabrication of biosensors with enhanced sensitivity.

## 1. Introduction

Owing to its unique structure, and chemical and optical properties, graphene and its derivatives have been employed for the detection of biomolecular and cell units [1,2,3,4,5]. Graphene oxide (GO), in particular, has abundant oxygen-containing functional groups, good water dispensability, and friendly biocompatibility, that make it suitable for many applications. Graphene-based field-effect transistors are often fabricated for detecting biomolecules, including DNA, protein, and bacterium [6,7,8]. For example, a graphene oxide-based immuno-biosensor was fabricated by Seo et al. for pathogen detection, through measuring the fluorescence quenching of GO [9]. Wang et al. used antibody-modified reduced graphene oxide (RGO) films to detect circulating tumor cells with extreme sensitivity [10]. Furthermore, a large number of surface plasmon resonance (SPR) sensors with enhanced sensitivities, based on graphene and its derivatives, have been reported during the past few years [3,11,12,13,14]. Under total internal reflection, graphene exhibited a greater reflection of transverse magnetic (TM) waves, when compared to transverse electric (TE) waves, known as a polarization-dependent absorption effect of graphene [15]. A sandwiched graphene structure was used to describe and investigate this phenomenon. In this structure, a graphene layer was sandwiched between a high-index medium (refractive index n_1_) and a low-index medium (refractive index n_2_). Recently, graphene-based refractive index optical sensors with high sensitivities and resolution, based on the polarization-dependent absorption of graphene under total internal reflection, have been reported [15,16]. These sensors were employed for distinguishing cancer cells from normal cells [17], investigating the dynamical gas parameters [18], and detecting NO_2_ gas [19]. Biomolecular interactions often resulted in changes in the refractive index, and SPR sensors were usually used to investigate biomolecular interactions, owing to their excellent performance, including real-time, label-free, and high sensitivity and resolution [20,21,22,23,24]. Because the polarization-dependent absorption of graphene was sensitive to changes in the refractive index, it was possible to detect biomolecules through polarization-dependent absorption of graphene under total internal reflection. However, the flat surface of RGO, fabricated by means of spin-coating, does not benefit the binding of biomolecules, due to the fact that biomolecules and cells prefer binding on the thick and wrinkled surfaces of graphene, rather than those which are thin and flat [6,7].

This study reports on the use of an RGO microshells (RGOM) optical biosensor, suitable for the determination of goat anti-rabbit IgG. The RGOM was fabricated by combining high-temperature reduction and self-assembly of polystyrene (PS) microspheres. Under total internal reflection, the RGOM also showed characteristics of polarization-dependent absorption, which was similar to that of RGO. When compared to RGO, RGOM could immobilize more biomolecules and thus yield sensors with an enhanced performance. The latter is of great importance for biosensing, especially for intensity-modified biosensors.

For a proper comparison, the RGO-based biosensor was also fabricated and functionalized, using the exact same procedure as the one used for the RGOM-based biosensor.

## 2. Experimental Study

### 2.1. Materials

Polystyrene microspheres (2 μm) were purchased from AlfaAesar Chemical Co., Ltd., Shanghai, China. The Goat anti-Rabbit immunoglobulin G (IgG), Rabbit immunoglobulin G (IgG), anti-Immunoglobulin M (IgM), and Rabbit IgG-FITC, were received from Beijing Biosynthesis Biotechnology Co., Ltd. Bovine serum albumin (BSA) and glycine were from Tianjin Unite Stars Biotech Co., Ltd. (Tianjin, China). Phosphate Buffer solution (PBS) was purchased from Beijing Zhongshan Golden Bridge Biotechnology Co., Ltd. (Beijing, China). All solutions were prepared with distilled water. PBS (0.01 mol/L, pH = 7.4) was diluted using deionized water, and Goat anti-Rabbit IgG, Rabbit IgG, and Bovine IgM, were diluted using PBS. 1-Ethyl-3-(3-dimethyllaminopropyl) carbodiimide hydrochloride (EDC, 0.4 mol/L) and *N*-Hydroxysuccinimide (NHS, 0.1 mol/L) were received from Aladdin (Shanghai, China). Glycine (0.01 mol/L, pH = 2.0) was diluted using deionized water, and the pH of the resulting glycine solution was then adjusted to 2.0 using hydrochloric acid.

### 2.2. Fabrication of RGOM

**Figure 1:**
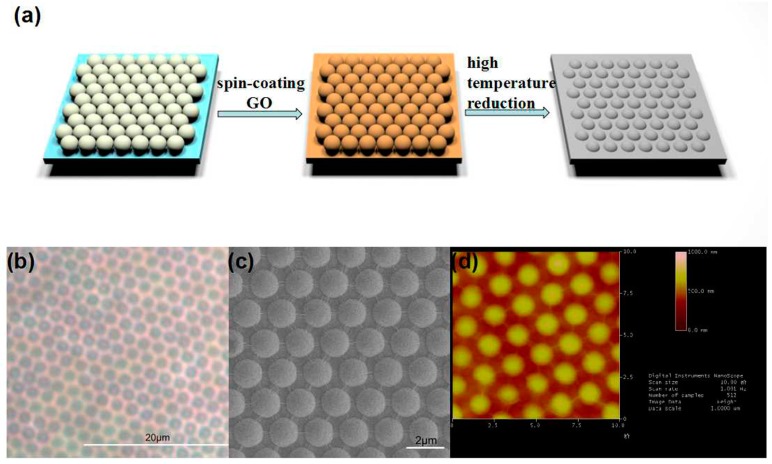
(**a**) Schematic representation of the RGOM fabrication process. First, a large-scale and closely packed monolayer of PS microspheres was formed on the precleaned SiO_2_ substrate, through self-assembly. An aqueous dispersion of GO was then spin-coated onto the surface of PS/SiO_2_. Finally, the GO film on the PS/SiO_2_ structure was reduced at a high temperature, resulting in an RGOM film; (**b**) Optical microscopy (OM)images of RGOM; (**c**) Scanning electron microscopy (SEM) images of RGOM; (**d**) Atomic force microscopy (AFM) images of RGOM.

GO was prepared from natural graphite using the modified Hummer’s method [25], and RGOM was prepared as schematically displayed in Figure 1a. Firstly, PS microspheres with diameters of ~2 μm were used to fabricate a large-scale and closely packed monolayer of PS microspheres, through self-assembly on a pre-cleaned SiO_2_ substrate (Figure S1). Then, GO dispersed in water (5 mg/mL) was spin-coated (2500 rpm, 30 s, 2 times) onto the PS/SiO_2_ surface. Due to shadow effects, GO caps were also formed on the PS spheres, to produce microshells with PS cores. Finally, the GO film on PS/SiO_2_ was annealed at 800 °C under argon (95%) and hydrogen (5%) atmospheres (volume ratio), to yield RGOM. This experimental strategy provided an efficient way of growing ordered graphene microstructures over a large area.

### 2.3. Fabrication of RGOM-Based Sensor

Poly(dimethylsiloxane) (PDMS, Sylgard 184, Dow-corning) was used to fabricate the flow cell, due to its biocompatibility and chemical stability. The pre-fabrication template was then covered by PDMS pre-polymer (10:1, mass ratio) and placed in a 75 °C oven for 2 h. Subsequently, the flow cell was peeled off the template. Oxygen plasma was used to clean the redundant RGO, using a masking method. Finally, the binding between the flow cell and RGO/quartz was achieved through oxygen plasma etching, which enhanced oxygen-containing functional groups on the top surface of the RGO.

### 2.4. Pretreatment and Modification of RGOM Sensing Surface

Firstly, PBS (0.01 mol/L, pH = 7.4) was used to flush the flow cell, and then a mixed solution containing EDC (0.4 mol/L)/NHS (0.1 mol/L) was injected into the surface of the RGOM, through the flow cell, in order to activate the oxygen-containing functional groups. After 30 min, PBS was injected into the surface of the RGOM. Subsequently, rabbit IgG (1 mg/mL) was injected into the flow cell and antibody was immobilized onto the surface of the RGOM. The flow cell was then washed again with PBS, and BSA (10 mg/mL) was injected into the flow cell in order to block the formation of nonspecific bindings. For comparative purposes, PBS was injected as a baseline solution.

### 2.5. Experimental Apparatus

**Figure 2:**
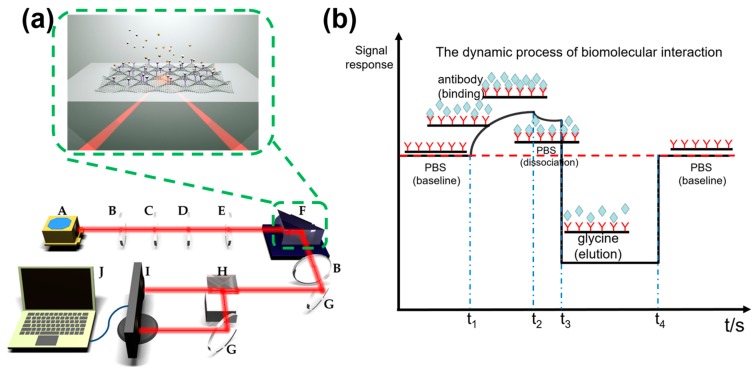
(**a**) Schematic representation of the RGOM-based optical sensing apparatus, the inset of (**a**) shows the core device of the sensor (RGOM/prism). A, laser; B, aperture; C, polarizer; D, half-wave plate; E, lens; F, flow cell/RGOM/prism; G, mirror; H, polarization beam splitter; I, balanced detector; J, computer; (**b**) The dynamic process of the biomolecular interactions process based on the RGOM optical biosensor, which was similar to that of an SPR biosensor.

The measurements of changes in the polarization-dependent absorption of graphene, were recorded using our homemade equipment, shown in Figure 2a. First, light from a He–Ne laser (λ = 632.8 nm) was converted into linear polarized light, using a polarizer, which was then focused on the sample index-matched to a K9 prism substrate. The reflected light was then separated into transverse electric (TE) polarized light and transverse magnetic (TM) polarized light, using a polarization beam splitter. The power difference in the separated light was recorded and compared using a balanced photodetector.

### 2.6. Detection of Goat Anti-Rabbit IgG and Sensor Regeneration

Firstly, PBS was injected as the baseline solution. Subsequently, the goat anti-rabbit IgG solution was then injected into the flow cell, using the constant current pump at a flow-rate of 100 μL/min. The binding between rabbit IgG and goat anti-rabbit IgG induced changes in the polarization-dependent absorption of graphene, where the real-time measurements were recorded using a balanced detector. After the reaction had reached equilibrium, PBS was injected again. A solution of glycine (0.01 mol/L, pH = 2.0) was then employed to elute the goat anti-rabbit IgG, and PBS was used to wash the flow cell so that the signal could return to the baseline, indicating the successful regeneration of the sensor.

## 3. Results and Discussion

### 3.1. Detection Methodology

The RGOM-based optical sensing apparatus is schematically presented in Figure 2a. The inset of Figure 2a shows the core structure of the sensor, consisting of a flow cell, RGOM/quartz, and a prism. The graphene layer binding the rabbit IgG was sandwiched between a high-index medium (refractive index n_1_, quartz) and a low-index medium (refractive index n_2_, solution). The rabbit IgG was bound onto the RGOM surface and functioned as the biorecognition element to bind goat anti-rabbit IgG. When the target antibody (goat anti-rabbit IgG) was injected, the interaction between the antibody and the antigen on the surface of the graphene, induced changes in the refractive index and thus led to modifications of the polarization dependent absorption of graphene. Information relating to the antibody-antigen binding was obtained by measuring and comparing the difference in power between TE and TM polarized lights.

### 3.2. Characterization of RGOM and RGO

The OM image in Figure 1b shows the periodicity of RGOM, corresponding to the configuration defined by the PS microsphere film. The periodic gray color contrast in the SEM image of Figure 1c, clearly reveals the RGOM morphology. The surface topography of the RGOM was further characterized by AFM, and the results are shown in Figure 1d. The RGOM prepared using high-temperature reduction exhibited a periodic structure consistent with PS microsphere templates. The recorded period of the microshells was ~2 μm, which is consistent with the SEM results.

In order to demonstrate the decomposition of PS microspheres during high-temperature annealing processes, the RGOM was transferred into a PDMS substrate and SEM was used for characterization. Figure S2a confirms the decomposition of almost all of the present PS microspheres. These results were further corroborated by Raman spectroscopy, which depicted the decomposition of PS microspheres. As shown in Figure S3a, the Raman peak, characteristic of PS, vanished after being processed at high-temperature annealing. The as-grown RGOM and RGO were measured by Fourier transform infrared spectroscopy (FTIR), and optical transmittance methods and the associated results are shown in Figures S2b and S3b, respectively. A comparison of the data with those of Figure S2d depicted no obvious presence of pyrolytic carbon. The thickness of the RGO was estimated by atomic force microscopy. Furthermore, the average thickness was recorded, exhibiting a figure of ~6.6 nm (Figure S4).

### 3.3. The Immobilization of Rabbit IgG

**Figure 3:**
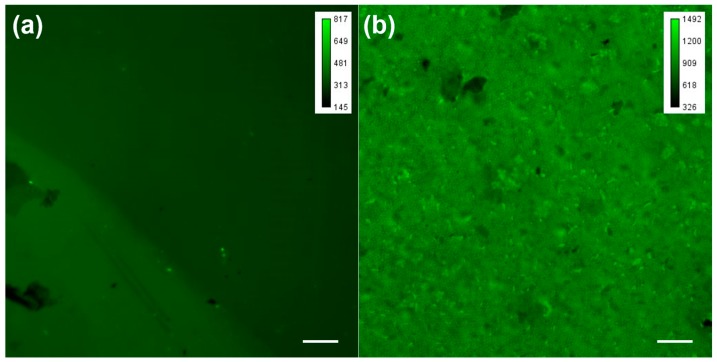
Fluorescent data of IgG tethering on both surfaces of RGO and RGOM. (**a**) Fluorescence IgG image of RGO; (**b**) Fluorescence IgG image of RGOM. Scale bar: 20 μm.

In order to determine the binding of rabbit IgG on the surface of the graphene, fluorescent rabbit IgG (1 mg/mL), which was diluted using the immunofluorescence staining antibody dilution buffer (Solarbio), was used, and the binding ability based on fluorescent intensity was then investigated by confocal microscopy, with an exposure time of 50 ms. These results were obtained under the same condition, by an inverted fluorescence/differential interference contrast microscope (Axio Observer D1, Carl Zeiss, Oberkochen, Germany), equipped with an electron multiplier CCD (DU-897, Andor, Belfast, UK). A comparison of Figure 3a,b revealed the evidently elevated fluorescence intensity of the RGOM surface, with respect to that of the RGO. This indicated that IgG preferred to bind to the surface of RGOM, rather than that of RGO. Two reasons might explain this: (i) the periodic microshells enhanced the specific surface area of graphene, so that more biomolecules could be functionalized into the surface of the RGOM; and (ii) periodic microshells could enhance the existence of wrinkles, and make it easier for bioreceptors to functionalize and modify the RGOM, than the RGO [5]. Hence, the RGOM was better suited to the detection of interactions between biomolecules in a solution, and biorecognition elements on the graphene surface.

### 3.4. Detection of Goat Anti-Rabbit IgG

**Figure 4:**
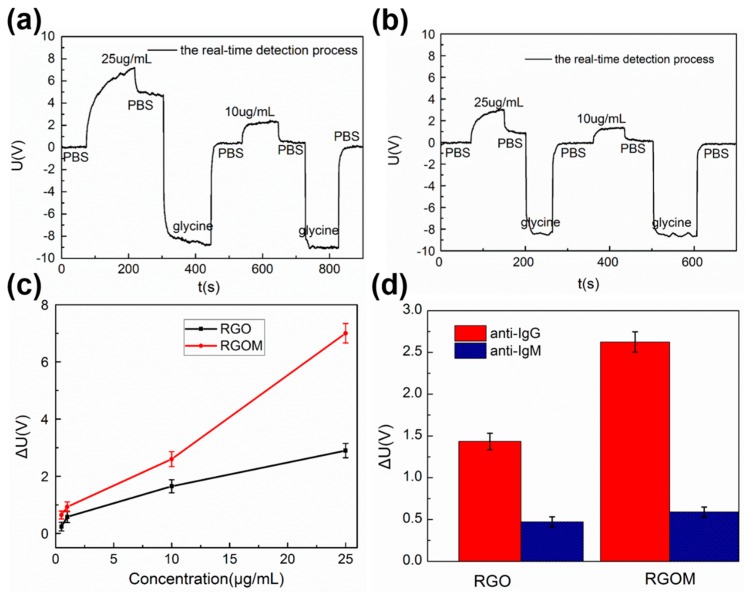
(**a**) Real-time measurement results of the RGOM-based biosensor; (**b**) Real-time measurement results of the RGO-based biosensor. The entire dynamic process was similar to that of an SPR-based biosensor, during which the sensor's response increased as the antibody concentration rose; (**c**) Comparative results of different concentrations of anti-IgG; (**d**) Specific detection ability of both the RGO-based and RGOM-based biosensors.

Figure 4 illustrates the real-time measurment results of the biosensors after fabrication, followed by the biochemical treatment. The binding, dissociation, and elution of the specific proteins, are important for investigating biomolecular interactions. In our experiments, a constant current injection pump was used to inject different solutions through the flow cell, at a flow-rate of 100 μL/min. Firstly, PBS was injected into the flow cell as the baseline solution. After injection of the antibody, the signal intensity began to rise as the antibody level increased. This demonstrated that the antibody dissolved in the solution was able to bind to the antigen, immobilized on the graphene surface. After another injection of PBS, some antibody molecules slowly dissociated from the antigen-RGOM surface, which led to a decrease in the signal intensity. This was further reduced as glycine was injected, but recovered to the baseline when PBS was further injected, clearly indicating that the antibody molecules thoroughly dissociated from the surface of the antigen-RGOM. Because the glycine solution has a lower refractive index than PBS, the signal decreased below the baseline. The entire process is schematically shown in Figure 2b, which is similar to the dynamic process of biomolecular interactions in SPR-based biosensors. Hence, the sensor looked like a potential candidate, except as a SPR sensor for the detection of biomolecules. Because goat anti-rabbit IgG could thoroughly be eluted from the rabbit IgG, the sensor could be employed for multiple uses, meaning lower costs per use, which is very important for practical applications. As shown in Figure 4a, the comparison between 10 μg/mL and 25 μg/mL injections of antibody, suggested that the signal issued from the antigen-antibody interactions was related to the concentration of the antibody solution, which increased as the concentration rose. For reference, the real-time measurements of an RGO-based sensor are shown in Figure 4b, showing a similar pattern to those of a RGOM-based sensor. However, the RGO-based sensor exhibited a weaker response than the RGOM-based biosensor under identical conditions, which was consistent with the fluorescence data. Figure S5 and Figure 4 also corroborate that the RGO with periodic microshells allowed the immobilization of more biomolecules, when compared to flat RGO. The polarization-dependent absorption of RGO, without binding of rabbit IgG molecules, was found to be stronger than that of RGOM under the same conditions, but the RGOM-based sensor containing rabbit IgG still showed a more satisfactory response to different concentrations of goat anti-rabbit IgG, than the RGO-based sensor.

Sensitivity is an important parameter to evaluate in biosensing. To examine the sensitivity of RGOM-based and RGO-based biosensors, different concentrations of goat anti-rabbit IgG were separately introduced into the flow cell at room temperature. The sensitivity of the sensor (ΔV), as a function of the concentration of goat anti-rabbit IgG (μg/mL), is shown in Figure 4c. It can be seen that the RGOM-based sensor exhibited a larger signal change than that of the RGO-based sensor, at different concentrations of antibody, under the same conditions. This further confirmed that periodic microshells were beneficial for immobilizing biomolecules onto the surface of graphene. A minimum concentration of 0.5 μg/mL goat anti-rabbit IgG solution might be distinguished for the RGO-based biosensor with a voltage change of about 0.23 V, which was much smaller than that of the RGOM-based biosensor with a voltage change of about 0.64 V. Because the sensor was based on intensity-modification, it was easily affected by other factors, such as the optical power and temperature. Larger response signals are of great importance, especially for the detection of low concentrations of biomolecules. This indicated that these sensors were highly reliable. Hence, the RGOM-based sensor was more suitable for detecting biomolecular interactions than the RGO-based sensor.

Chen et al. [26] compared the performance of graphene glass-based sensors (detection limit 0.1 μg/mL, response 0.1 V), using a commercial SPR sensor to detect the anti-IgG. They showed that a detection of 0.625 μg/mL was difficult to achieve for a commercial SPR sensor (angle-modified sensor). Compared to their results, the RGOM-based biosensor (detection limit 0.5 μg/mL, response 0.64 V) showed a satisfactory response towards goat anti-rabbit IgG, especially with the intensity-modified sensors. The larger detection signal obtained, indicated a higher accuracy, an aspect of great importance for practical applications.

### 3.5. Specific Detection

Specificity is an important parameter to evaluate in biosensing applications, which was verified using anti-Immunoglobulin M (10 μg/mL) as the mismatched protein introduced to the sensor by a similar procedure as goat anti-rabbit IgG. The response of the sensor is shown in Figure 4d for anti-IgM and anti-IgG with IgG. The data clearly revealed that the response to the mismatched anti-IgM was significantly smaller when compared to that obtained from complementary anti-IgG.

## 4. Conclusions

A novel method for fabricating RGO microshells and RGOM-based optical biosensors was developed, on the basis of polarization-dependent absorption of graphene under total internal reflection. Due to the presence of periodic microshells, the RGOM-based biosensor showed better detection of molecular interactions, when compared with the RGO-based biosensor. Moreover, the sensor exhibited similar dynamic processes of biomolecular interactions, which were often associated with SPR-based biosensors. The response of the biosensors increased as the antibody concentration rose. A number of antibodies or antigens could be detected using the sensors, by decorating the RGOM with selective antigen or antibody conjugates. This approach provides a novel effective route for the fabrication of graphene-based biosensors, suitable for the detection of different proteins during in vitro diagnostics.

## Supplementary Materials

The following are available online at http://www.mdpi.com/1424-8220/17/2/221/s1, Figure S1: Morphology and structure of the PS microsphere films, Figure S2: Scanning electron microscopy images and FTIR spectrums of RGO and RGOM, Video S3: Raman spectrums and transmittance of RGO and RGOM, Figure S4: Atomic force microscope image of RGO, Figure S5: Polarization-dependent absorption results of RGO and RGOM.
