# Supplementary material for: Microshell Arrays Enhanced Sensitivity in Detection of Specific Antibody for Reduced Graphene Oxide Optical Sensor
Source: Sensors (Basel). 2017 Jan 24;17(2):221. doi: 10.3390/s17020221 (PMC5336008; doi:10.3390/s17020221)
Supplement: Supplementary file 1 [file sensors-17-00221-s001.pdf]

Supplementary Materials: Microshell Arrays Enhanced Sensitivity in Detection of Specific Antibody for Reduced Graphene Oxide Optical Sensor

Wen-Shuai Jiang, Wei Xin, Shao-Nan Chen, Cun-Bo Li, Xiao-Guang Gao, Lei-Ting Pan, Zhi-Bo Liu and Jian-Guo Tian

Experimental Section

Preparation of PS microsphere arrays: The SiO_2 substrates ($20 \times 20 \times 1.5$ mm^3) were ultrasonically cleaned in acetone (10 min), alcohol (10 min), and rinsed with deionized water. A monolayer hexagonal close-packed arrays of monodisperse polystyrene (PS) spheres with diameters of ~2μm were formed on a pretreated quartz surface using the self-assembly process. The optical microscope, scanning electron microscope and atomic force microscope were used to investigate the PS microsphere arrays, and the results were shown in Figure S1.

Materials characterization: The surface morphologies of RGON were investigated using optical microscopy (Nikon ECLIPSE Ti-U), AFM (Nanoscope Dimension™ 3100), and SEM (LEO1530VP). The Raman spectra were obtained using a RENISHAW RM2000 Raman system equipped with a 514 nm laser source and 50× objective lens. The Fourier transform infrared spectroscopy (FTIR) data were collected with a NEXUS 870. The optical transmittance spectroscopy measurements were performed using a HITACHI U-4100 spectrophotometer. The fluorescence intensity was obtained using an inverted fluorescence/differential interference contrast microscope (Axio Observer D1, Carl Zeiss, Oberkochen, Germany) equipped with an electron multiplier CCD (DU-897, Andor, London, UK).

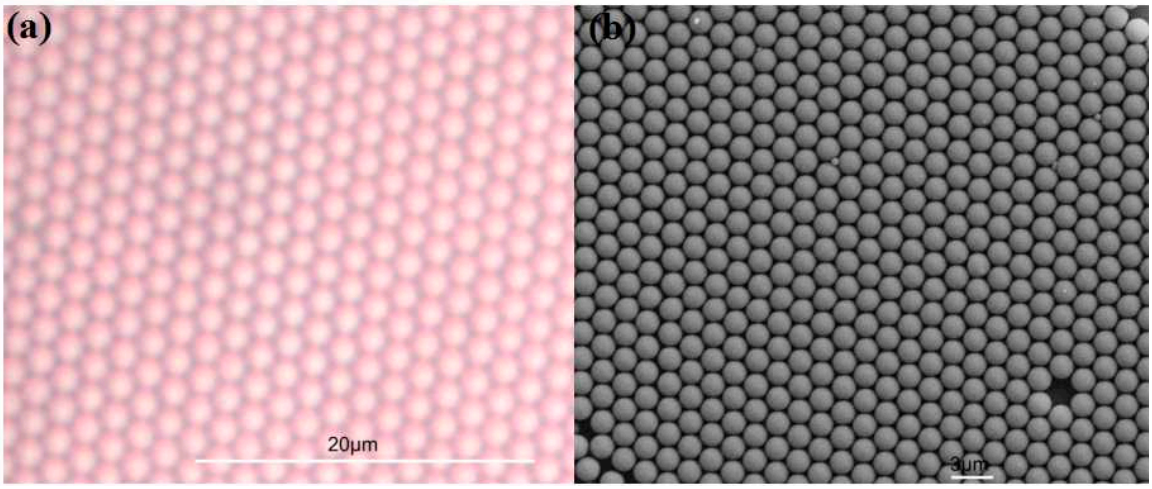

Figure 1. *Cont.*

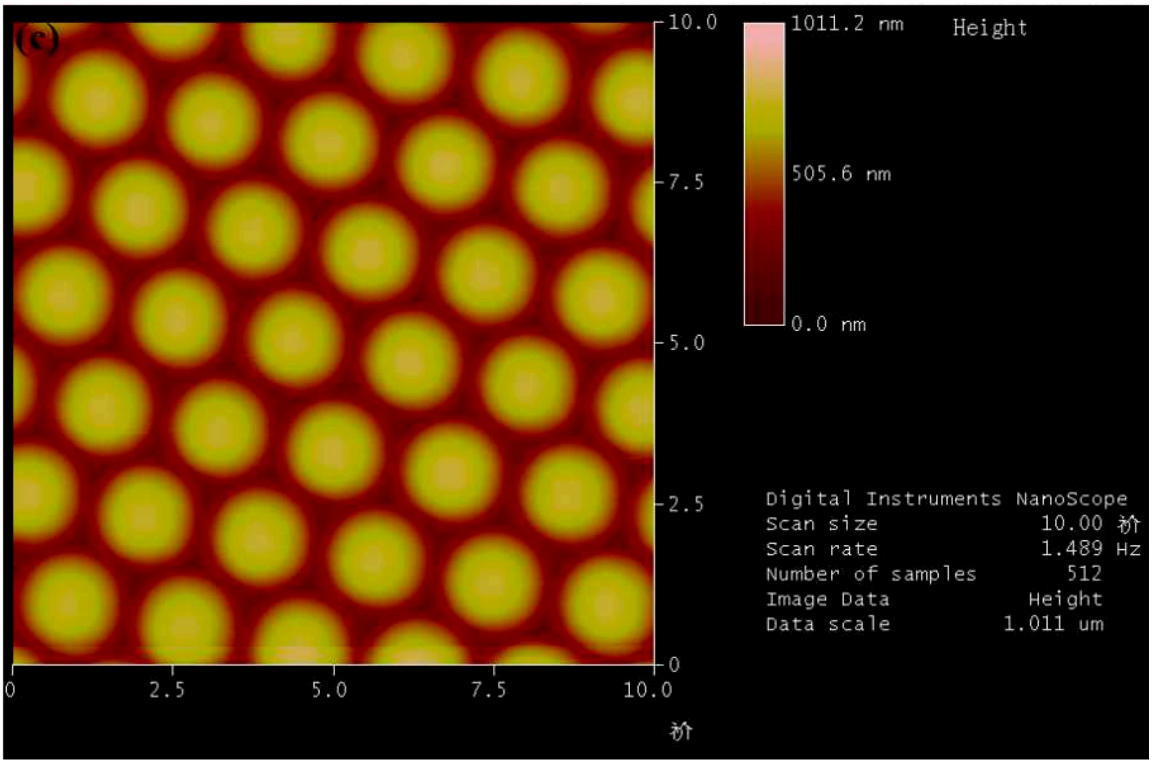

Figure S1. Morphology and structure of the PS microsphere films. (**a**) Optical microscopy image; (**b**) SEM image of the closely packed monolayer of PS nanospheres with a diameter of 2000 nm; (**c**) AFM image.

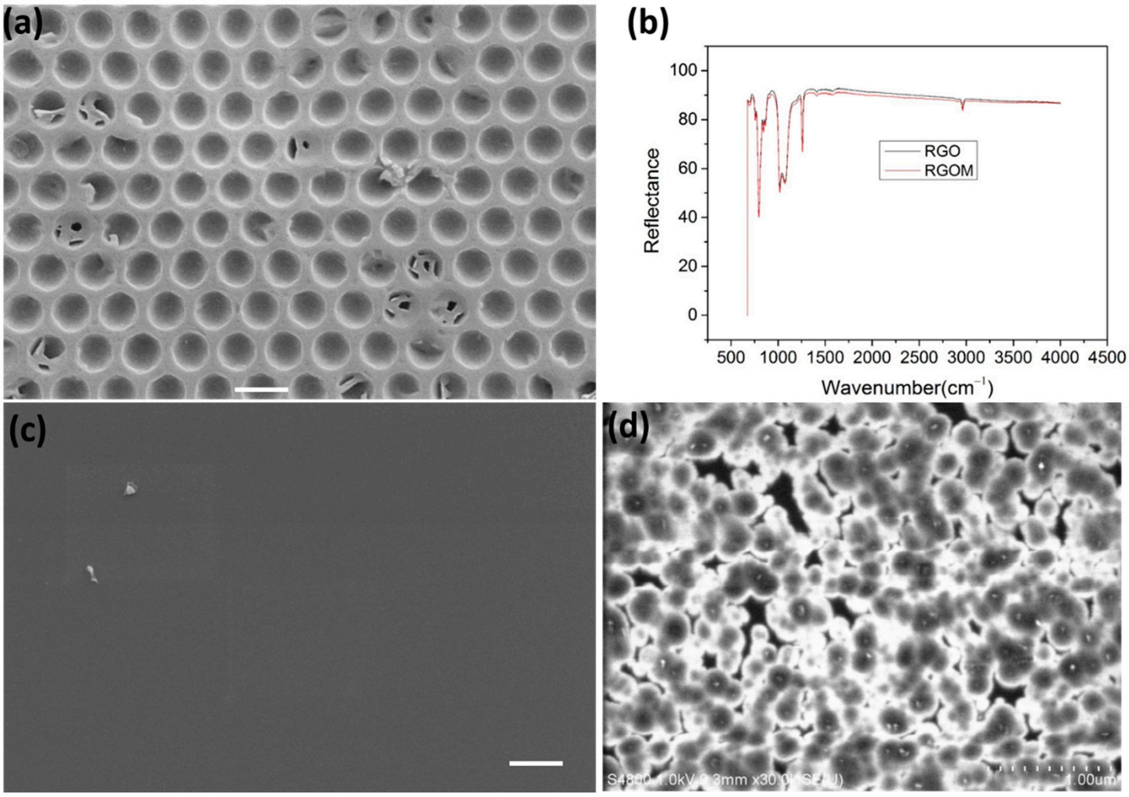

Figure S2. (**a**) Scaning electron microscope image of RGOM which was trasfered to PDMS substrate; (**b**) Fourier transform infrared spectroscopy (FTIR) of RGO and RGOM; (**c**) Scaning electron microscope image of RGO/SiO_2; (**d**) Scaning electron microscope image of amorphous carbon/SiO_2. The bar is 2 μm.

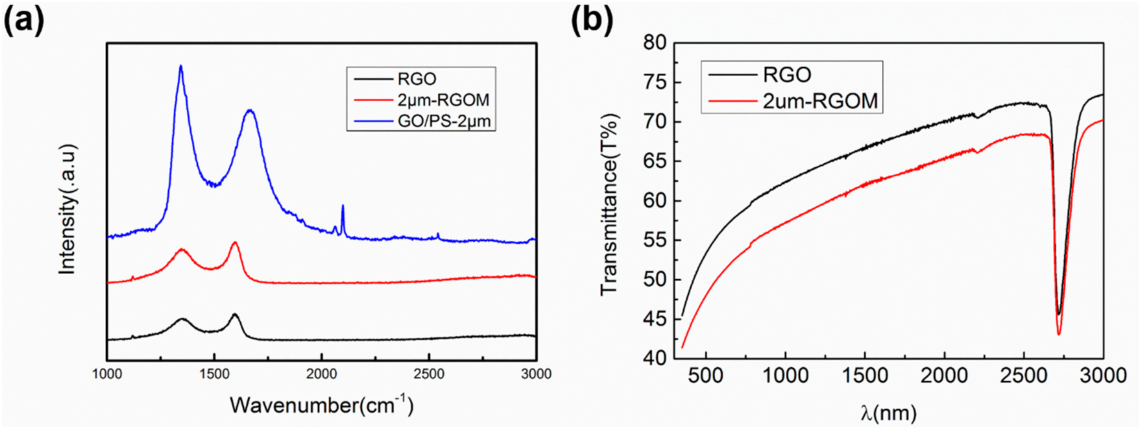

Figure S3. (**a**) Raman spectra of RGO and 2 μm -RGOM; (**b**) Optical transmittances of RGO and 2 μm –RGOM.

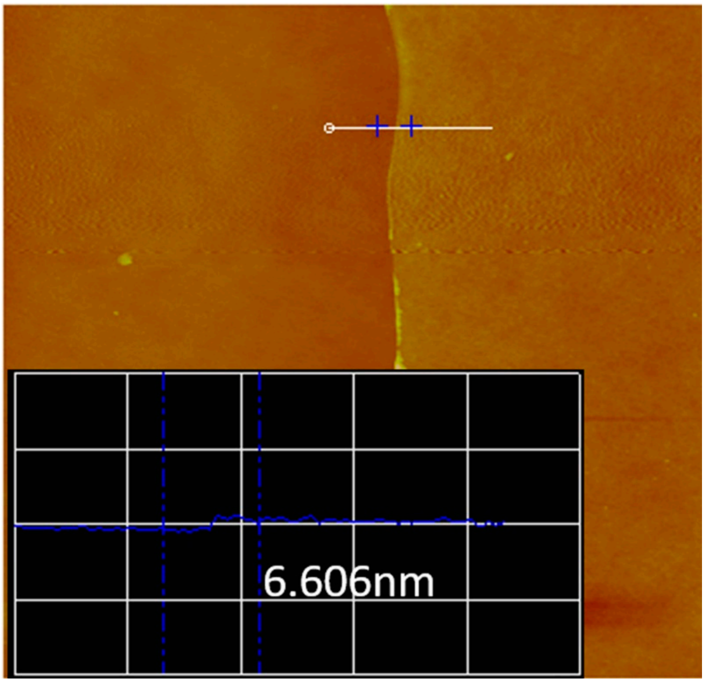

Figure S4. Atomic force microscope image of RGO. The result shows the thickness of RGO is 6.606 nm.

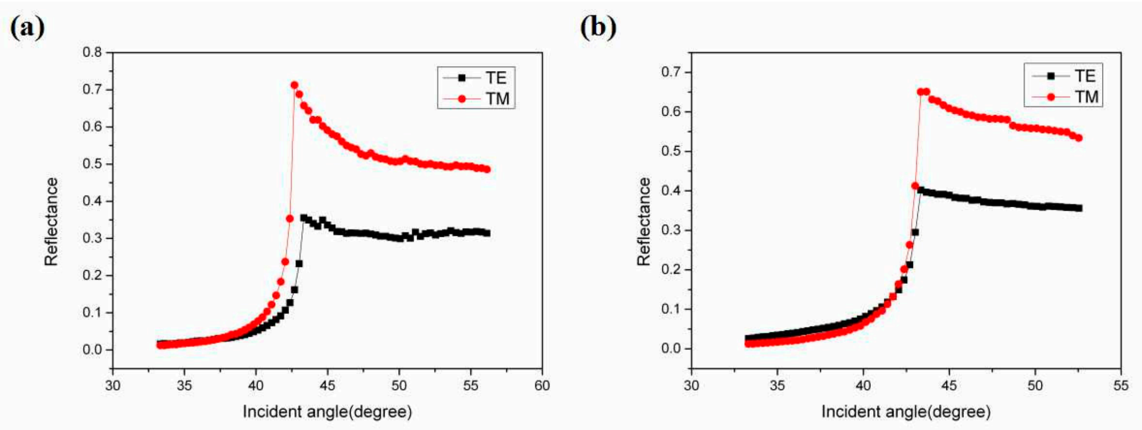

Figure S5. The experimental result for angular dependence of optical reflectance for TM and TE waves. (**a**) The experimental result of RGO; (**b**) The experimental result of RGOM. The polarization-dependent absorption of RGOM is similar with RGO.
